# Tobacco Consumption and Mental Health in the Canary Islands: A Quantitative Analysis

**DOI:** 10.3389/fpubh.2022.959442

**Published:** 2022-07-11

**Authors:** Imanol L. Nieto-González, M. Carolina Rodríguez-Donate, Ginés Guirao-Pérez

**Affiliations:** Departamento de Economía Aplicada y Métodos Cuantitativos, Universidad de La Laguna, San Cristóbal de La Laguna, Spain

**Keywords:** tobacco, mental health, multinomial logit, profiles, prevention

## Abstract

Although the detrimental health effects of tobacco, there has been scant research into determining comprehensive profiles to characterize individuals with a higher risk of smoking. This paper identifies such profiles and probabilistically quantifies the effects of certain traits on the decision to smoke in the Canary Islands. This characterization is approached from a broad and novel point of view for the region studied, given that the analysis not only studies sociodemographic characteristics, lifestyle and health, but also incorporates mental health. The results show that suffering from some type of psychopathology leads to a higher probability of tobacco consumption. Moreover, just as the promotion of a healthy lifestyle has a direct impact on tobacco consumption, the implementation of public policies that reduce the risks of suffering from a mental illness could also reduce the prevalence of smoking in the region and contribute to the design of more effective prevention strategies.

## Introduction

The tobacco epidemic is one of the world's greatest public health problems, causing more than eight million preventable deaths each year (1). Further exacerbating this problem is that smoking's effects on others are equally harmful, even if tobacco is not used directly. These figures, together with the existing evidence on the devastating health effects of tobacco, make tobacco prevention policies of vital importance and very relevant topic in the academic literature.

Efforts to reduce tobacco consumption are implemented through different national policies and regulatory frameworks. However, given the importance of this public health problem, the World Health Organisation (WHO), at a supranational level, promotes a range of measures. Recently, it presented the “WHO Report on the Global Tobacco Epidemic, 2021. Addressing new and emerging products” (2). This report is based on the Framework Convention on Tobacco Control (3) and the MPOWER strategy (4). It points out that alternative forms of smoking, such as smokeless tobacco or water pipes, also pose a high risk to health, as well as being a strong source of addiction. It also highlights the need for tobacco users to have help in coping with the process of quitting as an effective way to reduce consumption.

The need, therefore, to curb tobacco consumption means public policies aimed at this objective must be as effective as possible. These include legislative regulation to reduce tobacco consumption and regulating its sale, advertising or consumption in public places. If we analyse the effects of these types of laws in Spain, as these are applicable in the region under study (Canary Islands), we should highlight Law 28/2005, 26 December (5) and Law 42/2010, 30 December (6). Although not only in Spain (7, 8), there are many studies that demonstrate the usefulness of these regulations in terms of prevention (9). In addition, they have not had a negative collateral effect on other aspects, such as economic activity in the hospitality industry (10, 11), despite the total bans on smoking, thus making anti-smoking laws an even more useful instrument. However, although their effectiveness has been demonstrated, they have not been enough to reduce smoking rates, among other things, because the effect of these regulations seems more favorable in individuals from higher social classes (12). Thus, there is a clear need to continue working on regulations, since, in fact, it is this group that consumes the least tobacco (2). In the specific case of the Canary Islands, despite the positive effects generated by the above laws, “further progress must be made jointly in legislative, fiscal and social measures to reduce tobacco consumption” (13), especially as the effects of the laws have been diminishing over the years.

Moreover, according to the literature, prevention policies are also very important, beyond the approval of laws and taxation, especially those based on the specific characteristics that pose the greatest risk of tobacco use. Some studies point to the higher prevalence of tobacco consumption in men, as well as that age, increased income or a higher level of education discourage tobacco consumption (14–16). The prevalence in the case of smokeless tobacco is also higher for men, older, married and belonging to population groups with fewer resources (17). Know these profiles would allow policies to be properly targeted with better results, as the lack of targeting of groups with higher prevalence seems to be one of the main causes for their lack of success (18). However, to date, no studies have been found that, at a significant level of detail, characterize individuals on whom to focus strategies, which is the main line of study of this paper.

This work is confined to the Spanish region of the Canary Islands. This autonomous region, located 1,700 km from the Iberian Peninsula, is made up of eight islands, divided into two provinces. Its GDP per capita in 2021 was equivalent to 75.6% of the national GDP per capita. It is worth mentioning the differences in taxation between the archipelago and the rest of Spain, given that the islands not only have lower value added taxes, which means lower taxation on tobacco consumption, but also that the same tax on tobacco products that is applied throughout Spain is not applicable in the Canary Islands.

Among the total population of the Canary Islands aged 15 years and over, 22.3% use tobacco daily, compared to 22.1% at the national level (19). However, despite not being one of the Spanish regions with the highest proportion of tobacco users, (20) do rank as the region with the highest mortality rate attributed to tobacco use in women.

One characteristic of the archipelago is its status as an outermost region of the European Union, as well as the fact it is a region fragmented into different islands. Logically, this condition affects public health services (21). This means that the non-capital islands are affected by double insularity and are limited in their access to these services. As a result, the necessary support by health services in smoking cessation processes may be reduced. In terms of prevention and characterization of tobacco user profiles, there have been few studies conducted in the region. Among those available, (22) point out that tobacco consumption starts at an earlier age in the Canary Islands than in other regions. Likewise, in a study of adolescents, (23) conclude that some of the factors that explain tobacco consumption are related to the school or high school that individuals attend, supporting the idea proposed by other authors (24–26), where it is argued that the most effective prevention begins in schools. Finally, (27) conclude that the effect of “the group of friends, the consumption of alcoholic beverages and the lack of interest in studies” play an important role in the initiation of tobacco use, an idea that is in line with that expressed by authors such as (28), who emphasize the need to demystify and denormalize tobacco use in social and family settings.

On the other hand, one aspect that, until now, has not been considered in the literature framed in the Canary Islands as an explanatory factor of smoking is mental health. The following paragraphs introduce this idea and provide some of the evidence found on the relationship between smoking and mental health.

Mental health, within this work, is understood as psychological and emotional wellbeing, i.e., it is defined as something broader than the absence of mental disorders diagnosed by a doctor. This definition is in line with the WHO Constitution (29), which defines health as “a state of complete physical, mental and social wellbeing and not merely the absence of disease or infirmity.” Given the current importance of mental health, the WHO itself, as well as various states, are developing regulations that serve to protect and preserve the mental health of citizens. Specifically, in 2013, the World Health Assembly of the WHO approved the Mental Health Action Plan 2013–2020 (30), whose overall objective is to “promote mental health, prevent mental disorders, provide care, improve recovery, promote human rights and reduce mortality, morbidity and disability of people with mental disorders” (31).

In this regard, given the importance that mental health has been gaining, especially in recent years, several studies have examined its relationship with different socioeconomic variables (32–34) and with different personal situations, such as unemployment (35, 36) or economic crises (37–39). Regarding the relationship between mental health and tobacco consumption, the results of the work of (40) show that suffering from a mental disorder entails a higher risk of tobacco consumption. Along the same lines, (41) stress the need to implement specific plans aimed at preventing smoking among people with addictions or mental disorders. Zander Neves et al. (42) conducted a study among adolescents that led to the same conclusion, i.e., mental health, in addition to other characteristics such as family context, have a direct implication in the experimentation and use of tobacco among the group analyzed. Similarly, (43) point to the potential relationship between mental state and tobacco use, although they qualify the need to implement policies aimed at reducing the consumption of tobacco products in general, and not only focus on cigarettes.

Consequently, the aim of this work is to identify specific profiles of individuals who are more or less likely to smoke based on socio-demographic, lifestyle and health characteristics, with special attention to mental health. In short, the aim is to propose, in detail, which characteristics have a higher risk of consumption, using the probability associated with each characteristic as a classification factor. To achieve this objective, this work, in addition to the introduction, has a section dedicated to the data used, after which the methodological tool used, a multinomial logit model, is presented. This is followed by a section with the results of the estimated model, its main implications and conclusions.

## Materials and Methods

### Data

The data come from the 2015 Canary Islands Health Survey, conducted by the Canary Islands Institute of Statistics (44), which is the most recent source of health data available for the Autonomous Region of the Canary Islands. Specifically, the adult questionnaire was used for the population aged 16 and over, and the following variables were considered: socioeconomic variables of the individual, lifestyle habits, such as alcohol consumption or physical activity in their daily routine, and determinants of health status, like self-perceived health status or the presence of chronic diseases. With respect to chronic disease, 30 different diseases are included in the questionnaire. In this work, a dichotomous variable has been defined that indicates the presence of chronic disease and takes the value 1 if the individual has been diagnosed by a doctor with at least one of the chronic diseases included in the questionnaire and 0 otherwise.

In addition, obesity has been considered, as a way of capturing bad eating habits, quantified through the body mass index. Finally, an aspect of interest is the inclusion of the individual's mental health in the analysis to assess its impact on the decision to smoke.

The survey collects an individual's mental health through the GHQ-12 questionnaire that screens for possible current mental disorders (44). According to (45), this questionnaire, whose validity has been demonstrated for the Spanish population, can be used to assess psychological wellbeing and detect non-psychotic psychiatric problems. It is the same questionnaire that has been used in the National Health Survey of Spain for decades to assess mental health. The GHQ-12 is a reliable instrument as a one-dimensional test, it is a general construct of psychological distress that can be used as an initial screening (46). Specifically, it includes 12 items that, as indicated by (47), capture both the individual's psychological wellbeing and social functioning and ability to cope with worries. The questionnaire asks the individual whether during the last 30 days he or she has experienced, on a Likert-type scale, various issues related to mental health: able to concentrate, difficulty falling asleep, playing a useful part, decision-making ability, felt constantly under strain, couldn't overcome difficulties, able to enjoy day-to-day activities, able to face problems, feeling unhappy and depressed, loss of confidence, thinking of self as worthless, feeling reasonably happy. It is therefore an assessment of the individual's subjective mental state. In the Likert-type scale, 0–1–2–3, high scores indicate worse health. From the original scores, they are recoded in a dichotomous way: 0 (responses 1 and 2) and 1 (responses 3 and 4). In this way, the sum of the recoded scores ranges between 0 and 12 points.

To determine the existence or not of a mental disorder based on this questionnaire, various cut-off points have been used in the literature depending on the country [see the works cited by (47)]. In the case of Spain, (48) and (49) use scores above five as a cut-off point as indicators of psychopathology, transforming each item, which has four possible answers, into a dichotomous score. In (47) use as a cut-off point scores above the mean to indicate the presence of mental vulnerability. In the recent work by (50), they also use the GHQ-12, classifying individuals according to the absence of psychopathology (scores below 4), suspected psychopathology (between 5 and 6) and indicative of psychopathology (7–12).

In the present study, several indicators were considered to assess the absence of mental health or the presence of a disorder. Firstly, a score above 3 in the 12 items collected was considered as a cut-off point (51–53). According to this definition, 27.6% of the Canary Island population has mental health problems. Additionally, a second alternative was constructed considering the absence of good mental health to be those individuals whose score was above the mean plus one standard deviation (score above 5), representing 16.2% of the population. Finally, a score above 8 (score above the mean plus two standard deviations) was considered to identify individuals with a mental pathology. With this last, more restrictive criterion, the prevalence of mental health problems in the population is 7.8%.

The consideration of several indicators was motivated by the absence of a single indicator to assess mental health (54), as well as by the possible overestimation of mental health problems that, according to (51), the GHQ-12 produces, as it is a screening instrument that is more sensitive than specific. Taking this into account, mental health defined with the most restrictive criterion was included as an explanatory variable in the estimated model.

From a descriptive point of view, Table 1 presents the percentage of individuals who, within each explanatory variable, choose each of the smoking categories considered in this study. In relation to gender, the behavior of men and women differs, with men showing a higher proportion of smokers (30.3% of men and 23.3% of women) and ex-smokers (25.0% of men and 12.1% of women), while the majority of women are concentrated in the “never smoked” category (64.6%). The distribution according to age ranges also varies, with the intermediate age groups (26–45 years old and 46–65 years old) having the highest levels of smokers. However, although with differences in magnitude, the educational level categories behave similarly. Among the variables related to the individual's lifestyle, the positive relationship between alcohol consumption and smoking stands out at a descriptive level, since the higher the consumption of alcohol, the higher the concentration of smokers (from 18.3 to 39.1%). In terms of health, the contrast between having or not having a chronic disease does not seem to show a notable difference in the level of smokers; however, the gap within the category of ex-smokers reaches almost 10 percentage points. From the exploratory analysis of the mental health variable, it can be concluded that, among individuals with good mental health, the percentage of non-smokers is higher than among those with a psychopathology (56.7 and 50.9%, respectively), while the difference in the percentage of smokers is even greater, thus showing opposite and differentiated behaviors. It should be noted that, considering only smokers, 10.3% of them have mental health problems, while among the individuals in the sample who have never smoked, 7% declare they have some mental health problem.

**Table 1:** Tobacco use by characteristic and habits (%).

	**No smoking**	**Ex-smoker**	**Smoker**
**Gender**			
Women	64.6	12.1	23.3
Men	44.7	25.0	30.3
**Age**			
15–25	68.2	4.7	27.1
26–45	56.2	13.0	30.8
46–65	46.2	20.9	32.9
>65	68.3	21.8	9.9
**Family status**			
Married-partnered	57.5	21.2	21.3
**Level of education**			
Non-university studies	55.4	17.4	27.2
University studies	60.0	18.0	22.0
**Income level**			
Less than €500	48.8	14.3	36.9
500–2,000	57.5	17.5	25.0
More than €2,000	56.4	20.9	22.7
**Do you make physical activity part of your daily routine?**			
No	60.3	16.5	23.2
Yes	54.0	18.0	27.8
**Alcohol consumption**			
Never	68.1	13.6	18.3
Occasionally	54.3	17.2	28.5
Usually	33.2	27.7	39.1
**Body mass index**			
Obesity	58.9	21.6	19.5
**Self-perceived health status**			
Good	57.9	16.6	25.5
Fair	54.4	19.3	26.3
Bad	47.8	19.7	32.5
**Chronic disease**			
No	59.7	10.8	29.5
Yes	55.5	18.9	25.6
**Mental health**			
Not good	50.9	14.6	34.5
Good	56.7	17.7	25.6

### Methodology

In this paper, a discrete choice model is used to adequately capture the individual decision-making process from among a finite set of alternatives (55). A theoretical justification for these models is based on the Random Utility Theory approach, (56) in which it is assumed that individuals are rational and make decisions to maximize utility. Thus, the probability that an individual *i* chooses alternative *j* can be defined as the probability that this alternative has the highest utility among the set of possible alternatives (57). Utility of individual *i* for alternative *j* (*U*_*ij*_) can be expressed:


(1) $$\begin{array}{r} U_{ij}=V_{ij}+\varepsilon_{ij} \end{array}$$

Where *V*_*ij*_ is the systematic part, a set of explanatory variables (characteristics of the individual and attributes of the alternative), and ε_*ij*_ is the random part that includes the unobservable or measurable factors. Individual *i* will choose alternative *j*, if and only if:


$$\begin{array}{l} U_{ij}>U_{ik},\;\forall \;k\neq j \end{array}$$

Thus, the probability that individual *i* chooses alternative *j*, can be expressed as:


$$\begin{array}{l} P\left(Y_{i}=j\right)=P\left(U_{ij}>U_{ik}\right)=P\left(\varepsilon_{ik}-\varepsilon_{ij}<V_{ij}-V_{ik}\right)\;\forall \;k\neq j \end{array}$$

The number of alternatives from which the individual must choose, the ordered or unordered nature of the dependent variable, as well as the distribution assumed for the vector of disturbance terms will determine the model finally specified. In this paper, since more than two unordered alternatives are considered, a multinomial specification is chosen. Furthermore, assuming independent type I (Gumbel) extreme value distributions, the multinomial logit model is obtained where the probabilities of each alternative can be expressed:


$$\begin{array}{ll} P(Y_{i}=j) & =\frac{e^{x_{i}^{{}^{\prime }}\beta_{j}}}{1+\sum_{k=1}^{J}e^{x_{i}^{{}^{\prime }}\beta_{k}}}\;\;\;\;\;\;j=1,\ldots ,J\\ P(Y_{i}=0) & =\frac{1}{1+\sum_{k=1}^{J}e^{x_{i}^{{}^{\prime }}\beta_{k}}}\;\;\;\;\;j=0 \end{array}$$

Where *x*_*i*_ is the vector of explanatory variables, and β is the set of parameters to be estimated.

## Results

A multinomial logit model was estimated to explain the decision to use tobacco. The independence of irrelevant alternatives (IIA) was tested through the Hausman specification test (58). The dependent variable of the model is the consumption decision, which consists of three alternatives: no smoking (reference alternative), ex-smoker, smoker. The vector of explanatory variables, which includes individual, lifestyle and health characteristics, is made up of dichotomous variables that take the value 1 if the individual takes the characteristic in question and 0 otherwise. Table 2 presents the results of the model estimation. The table indicates the reference categories for each of the dichotomous variables considered in the model. However, to quantify the effect of each variable on the probability of each alternative, discrete changes must be obtained, since the parameters are not directly interpretable in magnitude or sign.

**Table 2:** Estimates of the multinomial logit model.

	**Ex-smoker**	**Smoker**
**Constant**	−3.8940^***^	−1.3379^***^
**Gender**		
Women (ref.)		
Men	0.9961^***^	0.4766^***^
**Age**		
15–25 (ref.)		
26–45	1.0695^***^	0.5274^**^
46–65	1.6487^***^	0.7354^***^
>65	1.3040^***^	−0.9016^***^
**Family Status**		
Single (ref.)		
Married-partnered	0.02725	−0.5187^***^
**Level of education**		
Non-university studies (ref.)		
University studies	−0.0565	−0.4608^***^
**Income level**		
Less than €500 (ref.)		
500–2,000	−0.0656	−0.3809^**^
More than €2,000	0.0777	−0.5226^**^
**Do you make physical activity part of your daily routine?**		
No	−0.2289^**^	−0.1258
Yes (ref.)		
**Alcohol consumption**		
Never (ref.)		
Occasionally	0.4338^***^	0.5998^***^
Usually	1.0863^***^	1.3798^***^
**Body mass index**		
No obesity (ref.)		
Obesity	0.1144	−0.4625^***^
**Self-perceived health status**		
Good (ref.)		
Fair	0.2589^**^	0.2959^**^
Bad	0.5462^**^	0.7688^***^
**Chronic disease**		
No (ref.)		
Yes	0.6591^***^	0.2125^*^
**Mental health**		
Good (ref.)		
Not good	−0.1390	0.3262^*^

*N = 3,660 $\chi^{\text{2}}\left(\text{32}\right)=\text{744.47 (0.0000)}\;R_{MCF}^{\text{2}}=\text{0.1038}$*.

*Percentage of correct predictions = 60.1%*.

*Levels of significance*:

*** *P < 0.01*;

** *P < 0.05*;

* *P < 0.10*.

Discrete changes are defined as the difference between the probability of each alternative when the individual shows a certain characteristic and when not presenting it. From the discrete changes, calculated for each alternative and for each individual in the sample and then averaged, the effects of the different characteristics on the probability of choosing each alternative can be observed (Table 3). Gender differences are confirmed, with men being more likely to smoke than women. The value 0.0369 of the discrete change in smoker alternative indicates that this probability is 3.6 percentage points (pp) higher for men. Individuals aged between 26 and 65 are more likely to smoke than younger individuals. The higher the income level, the lower the individual's probability of smoking compared to individuals with an income of less than €500 that is the reference category (between 6.4 and 8.7 pp lower). If the individual has a university education, his or her probability of smoking is 7.2% lower than if he or she does not have one (see the negative sign of the discrete change for university studies). The educational level was considered as university studies opposed to another because it is the category that presents a differentiated behavior with respect to the rest of the levels. In relation to bad lifestyle habits, not engaging in physical activity in their daily routine decreases the probability of smoking, and alcohol consumption, especially regular consumption, is positively associated with smoking (20.1% more likely to smoke than an individual who does not consume alcohol). With regard to the result for physical activity, it should be noted that this variable refers to habitual routine activity, i.e., physical activity carried out during the normal working day, study or, for example, housework. This definition means that its interpretation has more to do with daily routine than with the intention to do sport, so that the sign it takes could be explained by a sedentary lifestyle. This result could also be explained by the negative effect of a higher level of education and income on the probability of smoking, as these characteristics are usually associated with people who have jobs that tend to be sedentary.

**Table 3:** Discrete changes of the multinomial logit model.

	**No smoking**	**Ex-smoker**	**Smoker**
**Gender**			
Men	−0.1544^***^	0.1175^***^	0.0369^**^
**Age**			
26–45	−0.1620^***^	0.1287^**^	0.0333
46–65	−0.2463^***^	0.2021^***^	0.0442
>65	−0.0619	0.2539^***^	−0.1920^***^
**Family status**			
Married-partnered	0.0628^***^	0.0278^**^	−0.0906^***^
**Level of education**			
University studies	0.0602^**^	0.0122	−0.0724^***^
**Income level**			
500–2,000	0.0552^**^	0.0092	−0.0644^***^
More than €2,000	0.0528^*^	0.0337	−0.0866^***^
**Do you make physical activity part of your daily routine?**			
No	0.0360^**^	−0.0246^*^	−0.0114
**Alcohol consumption**			
Occasionally	−0.1131^***^	0.0303^**^	0.0829^***^
Usually	−0.2782^***^	0.0773^***^	0.2010^***^
**Body mass index**			
Obesity	0.0433^**^	0.0366^**^	−0.0799^***^
**Self-perceived health status**			
Bad	−0.1476^***^	0.0340	0.1136^***^
Fair	−0.0605^**^	0.0210	0.0395^**^
**Chronic disease**			
Yes	−0.0799^***^	0.0699^***^	0.1003
**Mental health**			
Not good	−0.0331	−0.0326	0.0656^**^

*Levels of significance*:

*** *P < 0.01*;

** *P < 0.05*;

* *P < 0.10*.

Regarding the obesity variable, the discrete changes indicate that this characteristic discourages consumption, since it increases the probability of not smoking (+4.33 pp) and reduces that of smoking (−7.99 pp). Although, a priori, it might be expected that the accumulation of bad habits would also include a potentially bad diet, given the characteristics of the sample, the result is consistent. In the sample, specifically, the second group with the highest percentage of obese people in descriptive terms is the over-65s, who in turn have the lowest percentage of obese smokers (4% compared with more than 20% in the other groups). The latter could explain the negative sign of the discrete change in this variable, bearing in mind that, in addition, by medical prescription, the over-65s with obesity should stop smoking (a group with more ex-smokers) to avoid extra risk factors in addition to their age and Body Mass Index.

In relation to health, the presence of chronic disease or the presence of fair or poor health have a greater impact on the likelihood of smoking (positive discrete changes). In the case of chronic illness, a higher probability of smoking on a daily basis may be related to the definition of the variable itself. In the survey, from the list of long-term or chronic diseases, some are considered, such as allergies, skin problems, cataracts, chronic constipation or hemorrhoids, which may not necessarily influence the individual to quit smoking.

In terms of mental health, individuals with some psychopathologies are 6.5% more likely to smoke than individuals without these pathologies (reference category). Although several models were estimated considering the mental health variable according to the three indicators mentioned in the description of the data, the best model turned out to be the one that incorporates the most restrictive criterion to determine the presence of psychopathology in individuals.

Table 4 presents the probabilities predicted by the model for individuals in the sample identified with and without some form of mental psychopathology. The probability of smoking for an individual with good mental health is 25.56%, while this probability is 34.49% for an individual who has some mental problem (varies by almost 9 percentage points). These results corroborate the importance of considering mental health as a risk factor when designing tobacco prevention and control policies more effective for this segment of the population and they are in consonance with other studies (59, 60).

**Table 4:** Predicted probabilities for each alternative by mental health.

	**No smoking**	**Ex-smoker**	**Smoker**
Not good mental health	0.5087	0.1463	0.3449
Good mental health	0.5669	0.1776	0.2556

Moreover, sub-sample multinomial models were also estimated for different individuals according to their reported mental health, i.e., for individuals with or without psychopathology. However, the results obtained showed worse fits, in terms of comparison with the full-sample models, so this proposal was discarded.

We also considered including a variable for affective and personal support as an explanatory factor for the decision to smoke. This would assess the extent to which having greater social support, either from family or friends, may influence an individual's smoking behavior. However, this variable was not significant in the decision to use tobacco.

It is also interesting calculating the probabilities predicted by the model for the profiles of individuals most and least likely to make each smoking decision detected from the discrete changes obtained. Table 5 shows significant differences for each of the alternatives. For example, an individual with characteristics most likely to smoke have a probability of 78.88%, while the probability of individual least likely is lower than 1.5%. The notable differences highlight the need to take into account the profiles of individuals in the design of effective prevention policies.

**Table 5:** Predicted probabilities for each alternative.

	**No smoking**	**Ex-smoker**	**Smoker**
Individual most likely	0.9489	0.6533	0.7888
Individual least likely	0.0609	0.0106	0.0122

## Conclusions

The aim of this work is to identify the profiles of individuals with a higher prevalence of smoking in the Canary Islands. We not only characterize individuals with a higher propensity to smoke, but also for non-smokers or ex-smokers. The analysis carried out, through the estimation of a multinomial logit model, allows us to quantify probabilistically the effects of individuals' socio-economic traits, as well as their life habits and health, both physical and mental, the latter understood as psychological and emotional wellbeing. Precisely, the consideration of the mental health indicator is one of the contributions of the work, not only because of the interest of incorporating mental health as an explanatory variable, a novel aspect in studies of this type, but also because of the difficulty it entails, as there is no consensus in the literature on how to define this indicator. Among the main results found, the positive relationship between greater tobacco consumption and the presence of some mental psychopathology stands out, revealing the importance of implementing policies of promotion and education on not only physical but also mental health care. On the other hand, there could be a multiplier effect on consumption for those individuals of male gender, with intermediate ages, low-income level, non-university studies, who consume alcohol frequently and have a poor health status, since these traits have a positive influence on smoking. In the case of ex-smokers, the traits that generated a greater propensity to smoke are male, over 65 years of age, with a university education, high income level, who consumes alcohol, no psychopathology and poor physical health, both in terms of their self-perceived state of health and the presence of chronic illness or obesity. However, the profile of the individual most likely to be a non-smoker is that of a young woman, under 26 years of age, with a university education, high income, who does not routinely engage in physical activity (associated with jobs requiring less physical effort), who does not consume alcohol and is in good physical and mental health.

The relevance of characterizing in detail the profile of individuals who use tobacco, but also of non-users, as well as ex-smokers, is key when defining prevention strategies that are not limited to regulation, but also direct the focus of policies toward those individuals who have a higher prevalence of consumption. Likewise, the analysis is particularly interesting for the geographical area chosen, given its status as an outermost region. Therefore, there is an added difficulty in designing prevention control strategies by the health service, as well as the differences in terms of legislation and taxation compared to the rest of the country, as far as tobacco is concerned, and the incidence of the habit in the young population of the Canary Islands. Based on the results found, regardless of the exact quantification of the incidence of each of the characteristics analyzed on the probability of smoking, it seems necessary to direct policies to discourage consumption toward individuals who, based on the profiles found, are more likely to smoke. Specifically, education should be encouraged at an early age, starting in schools, discouraging smoking, so that young people who do not go on to higher education and who may possibly have a lower paid job, requiring greater physical effort, with the consequent impact on their psychological and mental wellbeing, do not take up the habit. It is also concluded that the simultaneous implementation of prevention policies that preserve mental health and promote the reduction of alcohol consumption among the population, while at the same time implementing smoking prevention plans, may make the latter more likely to be successful.

As lines of future research, from a methodological point of view, we intend to explore the consideration of the heterogeneity associated with individuals' decisions through the specification and estimation of mixed models. From the economic point of view, it would be useful to study the criterion for determining individuals according to their state of mental health and to carry out the analysis in successive periods. Finally, it would be valuable to compare the profiles identified with those of other outermost regions in order to detect whether there are similar behavioral patterns. In addition, it is of interest to analyze the differences that, in terms of the study carried out, may occur between the different Autonomous Communities or regions of Spain, including in the analysis characteristics of the analyzed territory.

## Data Availability Statement

The data analyzed in this study is subject to the following licenses/restrictions: The anonymized microdata must be requested from the Instituto Canario de Estadística. Requests to access these datasets should be directed to http://www.gobiernodecanarias.org/istac/.

## Author Contributions

IN-G contributed to conceptualization, investigation, data curation, formal analysis, methodology, validation, writing-original draft, and writing-review and editing. MR-D contributed to conceptualization, investigation, data curation, formal analysis, methodology, validation, writing-original draft, writing-review and editing, and supervision. GG-P contributed to conceptualization, data curation, methodology, writing-original draft, writing-review and editing, and supervision. All authors contributed to the article and approved the submitted version.

## Funding

This work was co-financed by Agencia Canaria de Investigación, Innovación y Sociedad de la Información de la Consejería de Economía, Conocimiento y Empleo, and by Fondo Social Europeo (FSE) Programa Operativo Integrado de Canarias 2014–2020, Eje 3 Tema Prioritario 74 (85%).

## Conflict of Interest

The authors declare that the research was conducted in the absence of any commercial or financial relationships that could be construed as a potential conflict of interest.

## Publisher's Note

All claims expressed in this article are solely those of the authors and do not necessarily represent those of their affiliated organizations, or those of the publisher, the editors and the reviewers. Any product that may be evaluated in this article, or claim that may be made by its manufacturer, is not guaranteed or endorsed by the publisher.

## References

[1] Organización Mundial de la Salud. Declaración de la OMS: Consumo de Tabaco y COVID-19. (2020). Available online at: https://www.who.int/news/item/11-05-2020-who-statement-tobacco-use-and-covid-19 (accessed January 11, 2022).

[2] Organización Mundial de la Salud. Informe OMS Sobre la Epidemia Mundial del Tabaquismo, 2021. Hacer Frente a Productos Nuevos y Emergentes. (2021). Available online at: https://apps.who.int/iris/handle/10665/344224 (accessed January 11, 2022).

[3] Organización Mundial de la Salud. Convenio Marco de la OMS Para el Control del Tabaco. (2003). Available online at: http://apps.who.int/iris/bitstream/handle/10665/42813/9243591010.pdf?sequence=1 (accessed January 11, 2022).

[4] Organización Mundial de la Salud. Informe OMS Sobre la Epidemia Mundial del Tabaquismo, 2019. Ofrecer Ayuda Para Dejar el Tabaco. (2019). Available online at: https://www.who.int/publications/i/item/9789241516204 (accessed January 11, 2022).

[5] Ley 28/2005 de 26 de diciembre de medidas sanitarias frente al tabaquismo y reguladora de la venta el suministro el consumo y la publicidad de los productos del tabaco (BOE núm. 309, de 27 de diciembre de 2005). Available online at: https://www.boe.es/buscar/doc.php?id=BOE-A-2005-21261 (accessed June 28, 2022).

[6] Ley 42/2010 de 30 de diciembre por la que se modifica la Ley 28/2005 de 26 de diciembre de medidas sanitarias frente al tabaquismo y reguladora de la venta el suministro el consumo y la publicidad de los productos del tabaco (BOE núm. 318, de 31 de diciembre de 2010). Available online at: https://www.boe.es/buscar/act.php?id=BOE-A-2010-20138 (accessed June 28, 2022)

[7] Palali A Van Ours JC. The impact of tobacco control policies on smoking initiation in eleven European countries. Eur J Health Econ. (2019) 20:1287–301. 10.1007/s10198-019-01090-x 31485831 PMC6856042

[8] Serrano-Alarcón M Kunst AE Bosdriesz JR Perelman J. Tobacco control policies and smoking among older adults: a longitudinal analysis of 10 European countries. Addiction. (2019) 114:1076–85. 10.1111/add.14577 30868688 PMC6593806

[9] Pinilla J López-Valcárcel BG Negrín MA. Impact of the Spanish smoke-free laws on cigarette sales, 2000–2015. Health Econ Policy Law. (2019) 14: 536–52. 10.1017/S1744133118000270 30058518

[10] Caballero Hidalgo A Pinilla Domínguez J. Impacto sobre el consumo en bares, cafeterías y restaurantes de la modificación de la ley del tabaco española. Gac Sanit. (2014) 28:456–60. 10.1016/j.gaceta.2014.05.006 24950950

[11] García-Altés A Pinilla J Marí-Dell'Olmo M Fernández E López MJ. Economic impact of smoke-free legislation: did the Spanish tobacco control law affect the economic activity of bars and restaurants? Nicotine Tob Res. (2015) 17:1397–400. 10.1093/ntr/ntu346 25586775

[12] Pinilla J Abásolo I. The effect of policies regulating tobacco consumption on smoking initiation and cessation in Spain: is it equal across socioeconomic groups? Tob Induc Dis. (2017) 15. 10.1186/s12971-016-0109-4 28149259 PMC5273787

[13] Cuevas Fernández FJ Iglesias Girón MJ Rodríguez Pérez MC Ortiz Simarro S Cabrera de León A Aguirre-Jaime A. Evolución del tabaquismo según clase social en la población adulta de las Islas Canarias durante el periodo 2000–2015: seguimiento de la cohorte CDC-Canarias. Atención Primaria. (2020) 52:381–8. 10.1016/j.aprim.2019.05.007 31272849 PMC7256798

[14] Gallus S Luego A Liu X Behrakis P Boffi R Bosetti C. Who smokes in Europe? Data from 12 European countries in the TackSHS survey (2017–2018). J Epidemiol. (2021) 31:145–51. 10.2188/jea.JE20190344 32249267 PMC7813769

[15] Nargis N Thompson ME Fong GT Driezen P Ghulam Hussain AKM Ruthbah UH. Prevalence and patterns of tobacco use in Bangladesh from 2009 to 2012: evidence from international tobacco control (ITC) study. PLoS ONE. (2015) 10. 10.1371/journal.pone.0141135 26559051 PMC4641679

[16] Khandker NN Biswas T Khan ANS Hasib E Rawal LB. Socio-demographic characteristics and tobacco use among the adults in urban slums of Dhaka, Bangladesh. Tob Induc Dis. (2017) 15. 10.1186/s12971-017-0131-1 28484362 PMC5420145

[17] Mia MN Hanifi SMA Rahman MS Sultana A Hoque S Bhuiya A. Prevalence, pattern and sociodemographic differentials in smokeless tobacco consumption in Bangladesh: evidence from a population-based cross-sectional study in Chakaria. BMJ Open. (2017) 7. 10.1136/bmjopen-2016-012765 28122830 PMC5278241

[18] Inglés CJ Delgado B Bautista R Torregrosa MS Espada J P García-Fernández JM. Factores psicosociales relacionados con el consume de alcohol y tabaco en adolescentes españoles. Int J Clin Health Psychol. (2007) 7:403–20.

[19] Instituto Nacional de Estadística (INE). Encuesta Nacional de Salud. ENSE 2017. (2017). Available online at: https://www.ine.es/dyngs/INEbase/es/operacion.htm?c=Estadistica_C&cid=1254736176783&menu=resultados&idp=1254735573175 (accessed January 11, 2022).

[20] Rey J Pérez-Ríos M Santiago-Pérez MI Galán I Schiaffino A Varela-Lema L. Mortalidad atribuida al consumo de tabaco en las comunidades autónomas de España, 2017. Rev Esp Cardiol. (2022) 75:150–8. 10.1016/j.recesp.2020.10.018 33685853

[21] Abásolo Alessón I García Pérez L Aguiar Ibáñez R Amador Robayna A. Análisis del efecto de la condición de “doble insularidad” sobre la equidad en la utilización de servicios sanitarios públicos: el caso de las Islas Canarias. Investig Reg. (2008) 13:159–75.

[22] Henríquez Sánchez P Alonso Bilbao JL Beltrán Rodríguez R Doreste Alonso J. Tabaquismo en Gran Canaria. Consumo y actitudes en adolescentes. Gac Sanit. (2000) 14:338–45. 10.1016/S0213-9111(00)71491-9 11187451

[23] Pinilla J González B Barber P Santana Y. Smoking in young adolescents: an approach with multilevel discrete choice models. J Epidemiol Community Health. (2002) 56:227–32. 10.1136/jech.56.3.227 11854347 PMC1732090

[24] Fernández S Nebot M Jané M. Evaluación de la efectividad de los programas escolares de prevención del consumo de tabaco, alcohol y cannabis: ¿qué nos dicen los meta-análisis? Rev Esp Salud Publica. (2002) 76:175–87. 12092464

[25] Bartosh O Kozubovskyi R Shandor F Tovkanets O. Preventing the problema behavior: the case with Ukrainian vocational high school students. Int J Adolesc Youth. (2020) 25:464–78. 10.1080/02673843.2019.1674164

[26] Mélard N Grard A Robert P Kuipers M Schreuders M Rimpelä A. School tobacco policies and adolescent smoking in six European cities in 2013 and 2016: a school-level longitudinal study. Prev Med. (2020) 138. 10.1016/j.ypmed.2020.106142 32450162

[27] Caballero-Hidalgo A González B Pinilla J Barber P. Factores predictores del inicio y consolidación del consumo de tabaco en adolescentes. Gac Sanit. (2005) 19:440–7. 10.1016/S0213-9111(05)71394-7 16483521

[28] Kelly BC Vuolo M Frizzell LC Hernandez EM. Desnormalization, smoke-free air policy, and tobacco use among young adults. Soc Sci Med. (2018) 211:70–7. 10.1016/j.socscimed.2018.05.051 29894916

[29] Organización Mundial de la Salud. Constitución de la Organización Mundial de la Salud. (2006). Available online at: https://www.who.int/es/about/governance/constitution (accessed June 28, 2022).

[30] Organización Mundial de la Salud. Plan de acción sobre Salud Mental. 2013–2020. (2013). Available online at: https://apps.who.int/iris/bitstream/handle/10665/97488/9789243506029_spa.pdf (accessed January 11, 2022).

[31] Organización Mundial de la Salud. Salud Mental: Fortalecer Nuestra Respuesta. (2018). Available online at: https://www.who.int/es/news-room/fact-sheets/detail/mental-health-strengthening-our-response (accessed January 11, 2022).

[32] Bones Rocha K Pérez K Rodríguez-Sanz M Borrell C Obiols JE. Prevalencia de problemas de salud mental y su asociación con variables socioeconómicas, de trabajo y salud: resultados de la Encuesta Nacional de Salud de España. Psicothema. (2010) 22:389–95. 20667265

[33] Bacigalupe A Cabezas A Baza Bueno M Martín U. El género como determinante de la salud mental y su medicalización. Informe SESPAS 2020. Gac Sanit. (2020) 34:61–7. 10.1016/j.gaceta.2020.06.013 32900512

[34] Henares Montiel J Ruíz-Pérez I Sordo L. Salud mental en España y diferencias por sexo y por Comunidades Autónomas. Gac Sanit. (2020) 34:114–9. 10.1016/j.gaceta.2019.03.002 31053452

[35] Urbanos-Garrido RM Lopez-Valcarcel BG. The influence of the economic crisis on the association between unemployment and health: an empirical analysis for Spain. Eur J Health Econ. (2015) 16:175–84. 10.1007/s10198-014-0563-y 24469909

[36] Artacoz L Benach J Borrell C Cortés I. Unemployment and mental health: understanding the interactions among gender, family roles and social class. Am J Public Health. (2004) 94:82–8. 10.2105/AJPH.94.1.82 14713703 PMC1449831

[37] Stoyanova A Pinilla J. The evolution of mental health in the context of transitory economic changes. Appl Health Econ Health Policy. (2020) 18:203–21. 10.1007/s40258-019-00537-9 31761976

[38] Oliva J González López-Valcárcel B Barber Pérez P Peña-Longobardo LM Urbanos Garrido RM Zozaya González N. El impacto de la Gran Recesión en la salud mental en España. Informe SESPAS 2020. Gac Sanit. (2020) 34:48–53. 10.1016/j.gaceta.2020.05.009 32674864 PMC7358762

[39] Gili M López-Navarro E Castro A Homar C Navarro C García-Toro M. Gender differences in mental health during the economic crisis. Psicothema. (2016) 28:407–13. 10.7334/psicothema2015.288 27776609

[40] Rojas G Gaete J González I Ortega M Figueroa A Fritsch R. Tabaquismo y salud mental. Rev Med Chile. (2003) 131:873–80. 10.4067/S0034-98872003000800006 14558241

[41] Williams JM Ziedonis D. Addressing tobacco among individuals with a mental illness or an addiction. Addict Behav. (2004) 29:1067–83. 10.1016/j.addbeh.2004.03.009 15236808

[42] Zander Neves C Devicari Bueno C Pires Felden G Costa Irigaray M Fernanda Rivadeneira M Xavier Oenning NS. Tabaco en adolescentes escolares brasileños: asociación con salud mental y contexto familiar. Gac Sanit. (2018) 32:216–22. 10.1016/j.gaceta.2017.07.003 28923339

[43] King JL Reboussin BA Spangler J Ross JC Sutfin EL. Tobacco product use and mental health status among young adults. Addict Behav. (2018) 77:67–72. 10.1016/j.addbeh.2017.09.012 28965069 PMC5701872

[44] Instituto Canario de Estadística (ISTAC). Encuesta de Salud de Canarias. ESC 2015. Metodología. (2015). Available online at: http://www.gobiernodecanarias.org/istac/descargas/C00035A/ESC-2015-Metodologia.pdf (accessed January 11, 2022).

[45] Sánchez-López M Dresch V. The 12-item general health questionnaire (GHQ-12): reliability, external validity and factor structure in the Spanish population. Psicothema. (2008) 20:839–43. 18940092

[46] Pedrero-Pérez EJ Mora-Rodríguez C Rodríguez-Gómez R Benitez-Robredo MT Ordoñez-Franco A González-Robledo L. GHQ-12 in adolescents: contributions to the controversial factorial validity. Ann Psychol. (2020) 36:247–53. 10.6018/analesps.372721

[47] Villa IG Zuluaga C Restrepo LF. Propiedades psicométricas del Cuestionario de Salud General de Goldberg GHQ-12 en una institución hospitalaria de la ciudad de Medellín. Av Psicol Latinoam. (2013) 31:532–45.

[48] López-Castedo A Fernández L. Psychometric properties of the Spanish version of the 12-item general health questionnaire in adolescents. Percept Mot Skills. (2005) 100:676–80. 10.2466/pms.100.3.676-680 16060426

[49] Rocha K Pérez K Rodríguez-Sanz M Borrell C Obiols J. Propiedades psicométricas y valores normativos del general health questionnaire (GHQ-12) en población general española. Int J Clin Health Psychol. (2011) 11:125–39.

[50] Jorquera R Morales E Vega Al. Salud mental y apoyo social en habitantes de Copiapó, Chile, en el contexto de la COVID-19. Psicogente. (2021) 24:1–16. 10.17081/psico.24.46.4308

[51] Rocha K Perez K Rodríguez-Sanz M Borrell C Obiols JE. Prevalencia de problemas de salud mental y su asociación con variables socioeconómicas, de trabajo y salud: resultados de la Encuesta Nacional de Salud de España. Psicothema. (2010) 22:389–95. 20667265

[52] Basterra V. Evolución de la prevalencia de alto riesgo de trastornos mentales en población adulta española: 2006–2012. Gac Sanit. (2017) 31:324–6. 10.1016/j.gaceta.2017.01.004 28342634

[53] Brace O Duncan DT Correa-Fernández J Garrido-Cumbrera M. Association of sleep duration with mental health: results from a Spanish general population survey. Sleep Breath. (2022) 26:389–96. 10.1007/s11325-021-02332-0 34003436

[54] Salvador-Carulla L Salinas JA Martín M Grané M Gibert K Roca M. Indicadores Para la Evaluación de Sistema de Salud Mental en España. Madrid: Sociedad Española de Psiquiatría (2010) 10.13140/RG.2.2.19278.15689

[55] Greene WH. Econometric Analysis. 7th ed. London: Pearson Education Limited (2012). p. 1241

[56] Domencich T McFadden D. Urban Travel Demand: A Behavioral Analysis. Amsterdam: North-Holland (1975). p. 215.

[57] McFadden D. “Conditional analysis of qualitative choice models.” In: Zarembka P, editor. Frontiers in Econometrics. New York, NY: Academic Press. (1973).

[58] Hausman J McFadden D. A specification test for the multinomial logit model. Econometrica. (1984) 52:1219–40. 10.2307/1910997

[59] Guillén AI Marín C Panadero S Vázquez JJ. Substance use, stressful life events and mental health: a longitudinal study among homeless women in Madrid (Spain). Addict Behav. (2020) 103. 10.1016/j.addbeh.2019.106246 31838444

[60] López-Núñez C González-Roz A Weidberg S Fernández-Artamendi S. Sensibilidad a la ansiedad como factor de vulnerabilidad transdiagnóstico para el consume de tabaco: implicaciones clínicas y para el tratamiento. Adicciones. (2021) 33:85–94. 10.20882/adicciones.1549 33768255

